# Preclinical Development of a Fusion Peptide Conjugate as an HIV Vaccine Immunogen

**DOI:** 10.1038/s41598-020-59711-y

**Published:** 2020-02-20

**Authors:** Li Ou, Wing-Pui Kong, Gwo-Yu Chuang, Mridul Ghosh, Krishana Gulla, Sijy O’Dell, Joseph Varriale, Nathan Barefoot, Anita Changela, Cara W. Chao, Cheng Cheng, Aliaksandr Druz, Rui Kong, Krisha McKee, Reda Rawi, Edward K. Sarfo, Arne Schön, Andrew Shaddeau, Yaroslav Tsybovsky, Raffaello Verardi, Shuishu Wang, Timothy G. Wanninger, Kai Xu, Gengcheng J. Yang, Baoshan Zhang, Yaqiu Zhang, Tongqing Zhou, The VRC Production Program, Nadia Amharref, Christopher Barry, Boonchai Boonyaratanakornkit, Elizabeth Carey, Ria Caringal, Kevin Carlton, Naga Chalamalsetty, Adam Charlton, Rajoshi Chaudhuri, Mingzhong Chen, Peifeng Chen, Nicole Cibelli, Jonathan W. Cooper, Hussain Dahodwala, Marianna Fleischman, Julia C. Frederick, Haley Fuller, Jason Gall, Isaac Godfroy, Deepika Gollapudi, Daniel Gowetski, Joe Horwitz, Althaf Hussain, Vera Ivleva, Lisa Kueltzo, Yile Li, Venkata Mangalampalli, Gabriel Moxey, Sarah O’Connell, Aakash Patel, Erwin Rosales-Zavala, Elizabeth Scheideman, Nicole A. Schneck, Zachary Schneiderman, William Shadrick, Alison Vinitsky, Xiangchun E. Wang, Sara Witter, Yanhong Yang, Frank J. Arnold, Nicole A. Doria-Rose, Q. Paula Lei, Edward T. Ryan, Willie F. Vann, John R. Mascola, Peter D. Kwong

**Affiliations:** 1 Vaccine Research Center, National Institute of Allergy and Infectious Diseases, National Institutes of Health, Bethesda, 20892 MD USA; 2 Department of Biology, Johns Hopkins University, Baltimore, MD 21218 USA; 3 Electron Microscopy Laboratory, Cancer Research Technology Program, Leidos Biomedical Research Inc., Frederick National Laboratory for Cancer Research, Frederick, MD 21701 USA; 4 Massachusetts General Hospital, Boston, 02114 MA USA; 5 Center for Biologics Evaluation and Research, U.S. Food and Drug Administration, Silver Spring, 20993 MD USA

**Keywords:** Protein vaccines, HIV infections

## Abstract

The vaccine elicitation of broadly neutralizing antibodies against HIV-1 is a long-sought goal. We previously reported the amino-terminal eight residues of the HIV-1-fusion peptide (FP8) – when conjugated to the carrier protein, keyhole limpet hemocyanin (KLH) – to be capable of inducing broadly neutralizing responses against HIV-1 in animal models. However, KLH is a multi-subunit particle derived from a natural source, and its manufacture as a clinical product remains a challenge. Here we report the preclinical development of recombinant tetanus toxoid heavy chain fragment (rTTHC) linked to FP8 (FP8-rTTHC) as a suitable FP-conjugate vaccine immunogen. We assessed 16 conjugates, made by coupling the 4 most prevalent FP8 sequences with 4 carrier proteins: the aforementioned KLH and rTTHC; the *H. influenzae* protein D (HiD); and the cross-reactive material from diphtheria toxin (CRM197). While each of the 16 FP8-carrier conjugates could elicit HIV-1-neutralizing responses, rTTHC conjugates induced higher FP-directed responses overall. A Sulfo-SIAB linker yielded superior results over an SM(PEG)2 linker but combinations of carriers, conjugation ratio of peptide to carrier, or choice of adjuvant (Adjuplex or Alum) did not significantly impact elicited FP-directed neutralizing responses in mice. Overall, SIAB-linked FP8-rTTHC appears to be a promising vaccine candidate for advancing to clinical assessment.

## Introduction

The fusion peptide (FP) site of vulnerability on the HIV-1 envelope (Env) glycoprotein has recently been shown to be a promising vaccine target^1–3^. FP, a hydrophobic region of ~15 residues at the N terminus of the gp41 transmembrane glycoprotein, is an essential component of the HIV entry machinery^4^. FP embeds in the target cell membrane during the pre-hairpin intermediate stage of entry, where it serves to anchor the rearranging viral spike and to facilitate the merging of viral and cell membranes. The N-terminal portion of FP is solvent accessible and recognized by broadly neutralizing antibodies PGT151^5,6^, N123-VRC34.01^3^, and ACS202^7^. Because FP is a short linear peptide, it has low inherent immunogenicity due to its lack of helper T cell epitopes. Coupling peptides to highly immunogenic carrier proteins is a well-established approach for providing T cell help to peptide immunogens^8–11^. When the N-terminal 6–10 residues of FP are coupled to keyhole limpet hemocyanin (KLH), a standard protein carrier widely used in biotechnology, the resultant FP-KLH conjugate immunogens are able to induce broadly neutralizing FP-directed immune responses in mice, guinea pigs, and rhesus macaques^1,2,12^. Vaccine-induced FP-directed antibodies from mice or NHP neutralize up to 31% or 59%, respectively, of a cross-clade panel of 208 HIV-1 strains^2^.

**Figure 1:**
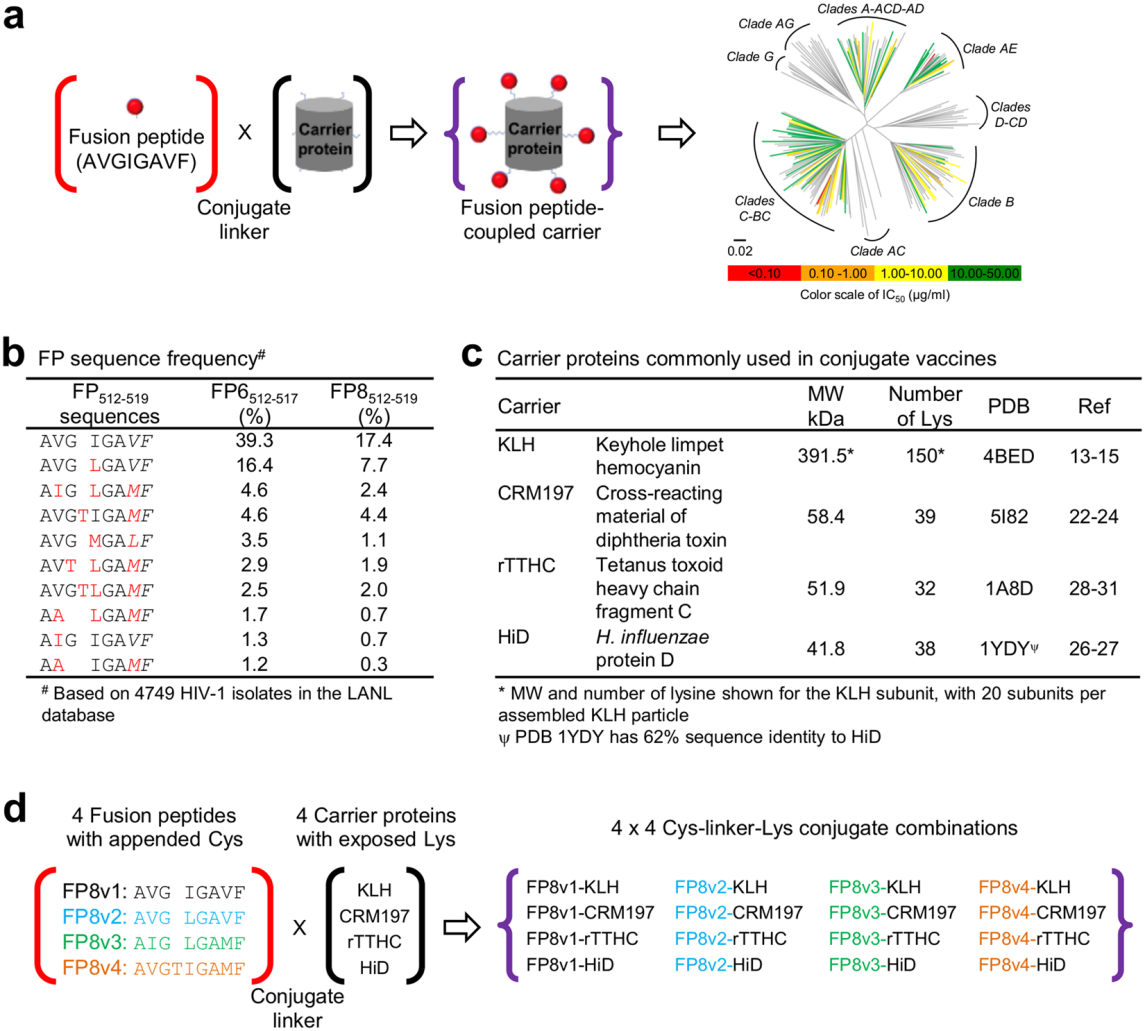
Strategy to assess different FP sequences and carrier proteins for their capacity to elicit HIV-neutralizing responses. (**a**) Schematic of experimental design. Fusion peptides are conjugated to carrier proteins (left) to elicit antibodies with cross-clade breadth (shown on a neutralization dendrogram for the FP-directed murine antibody vFP16.02^2^). (**b**) Prevalent FP sequences. FP sequence frequencies were calculated based on the N-terminal six or eight residues in the LANL HIV database^34^. (**c**) Properties of carrier proteins commonly used in conjugate vaccines. (**d**) Combination of 4 FP sequences with 4 carrier proteins to obtain 16 FP-carrier protein conjugates. Fusion peptides were synthesized with a C-terminal Cys to be coupled to the exposed Lys on the carrier proteins through a bi-functional linker. The four most prevalent FP sequences in (**b**) were chosen to have a wide coverage of HIV strains.

These results (illustrated in Fig. 1a) indicate FP coupled to a carrier protein to be a promising candidate immunogen. However, KLH is a multi-subunit metalloprotein derived from natural sources^13–15^ with both sequence and glycan heterogeneity, which pose manufacturing challenges to characterization and quality control. Moreover, native KLH is composed of subunit isoforms that assemble into a higher molecular weight form of ~8 MDa, which complicates standard manufacturing methods of filtration used for viral clearance. Together, these features make native KLH generally incompatible with standard processes of manufacturing, while the subunit form of KLH – not the assembled particle – has been used in clinical trials.

To date, a number of carrier proteins have been used in licensed conjugate vaccines and proven to be immunogenic and safe^16,17^. These include tetanus toxoid (TT)^18,19^, diphtheria toxoid (DT)^20,21^, modified cross-reacting material of diphtheria toxin (CRM197)^22–24^, meningococcal outer membrane protein complex (OMPC)^25^, and *Hemophilus influenzae* protein D (HiD)^26,27^. TT and DT have been used extensively in glycoconjugate vaccines since the early 1920s and are processed by formaldehyde to detoxify their highly lethal natural forms^17^. Several derivatives have been developed which preserve immunogenicity, eliminate toxicity, and improve manufacturability. A truncated, heavy chain-only, recombinant version of TT (rTTHC) has been developed as a clinically suitable carrier protein^28–31^, and a variant of diphtheria toxin from Corynebacterium diphtheriae C7 (β197) cultures has been developed (CRM197), which maintains T-helper epitopes and eliminates enzymatic activity and toxicity through a single glycine to glutamate substitution^32^. HiD is a highly conserved 42 kDa surface protein originally derived from non-typeable *H. influenzae*^33^, and is currently used as part of a carrier protein cocktail to deliver pneumococcal conjugate vaccines^26,27^.

One method of coupling peptides to carrier proteins is through a heterobifunctional crosslinker, with a sulfhydryl-reactive group to react with a cysteine side chain on the peptide and an amine-reactive group to react with surface-exposed lysine side chains on the carrier protein. Several such crosslinkers are available with varying spacer lengths, flexibility/rigidity, and hydrophilicity. Each of these linker characteristics can influence the immunogenicity of the resultant immunogen. Moreover, as the antigenic target of the HIV-1 FP site of vulnerability includes not only FP, but also the regions surrounding FP, the chemical character of the linker may affect the ability of FP-carrier conjugates to elicit FP-directed responses capable of neutralizing HIV-1.

Here we evaluated four commonly used carrier proteins for their ability to present prevalent HIV-1 FP sequences as immunogens in a mice model. We compared 16 different FP-protein conjugates and assessed the impact of a hydrophobic versus a hydrophilic crosslinker, of FP-carrier conjugation ratios, and of different adjuvants, Adjuplex and Alum, on the elicited immune responses. We found rTTHC to be a suitable carrier protein and Sulfo-SIAB to be a suitable linker for FP8-rTTHC conjugates. Overall, FP8-rTTHC with Sulfo-SIAB linker appeared to be a promising candidate immunogen for advancing to clinical assessment.

## Results

### Strategy to assess different carrier proteins and FP sequences for their capacity to induce HIV-1 neutralizing responses

To develop FP-carrier conjugates as immunogens for clinical evaluation, we analyzed the prevalence of FP sequences in the HIV LANL database^34^. The N-terminal 6 residues of FP account for 72% of the FP interface-buried surface area, when complexed with the broadly neutralizing FP-directed antibody N123-VRC34.01^3^, and we observed a similar focus on the N-terminal 6–8 residues of FP in FP-directed HIV-1 neutralizing antibodies elicited in mice and macaques^2,12^. The N-terminal FP residues are variably conserved, with a cumulative frequency of the top 4 most prevalent N-terminal sequences of FP6 (residues 512–517 of Env) utilized by ~65% of the HIV-1 isolates in the LANL database and the top 4 most prevalent N-terminal sequences of FP8 (residues 512–519 of Env) utilized by ~35% of the HIV-1 isolates in the LANL database (Fig. 1b). We coupled prevalent FP8-sequences (designated as FP8v1, FP8v2, FP8v3, and FP8v4) to commonly used carrier proteins^16^: rTTHC^28,35^, CRM197^22–24,36^, HiD^26^, and KLH^37,38^ (Fig. 1c and Supplementary Fig. S1). A total of 16 FP8-conjugate immunogens were made by coupling FP8 peptides, synthesized with an appended C-terminal Cys, with the heterobifunctional linker m-maleimidobenzoyl-N-hydroxysuccinimide ester (MBS), to exposed lysine residues on carrier proteins (Fig. 1d).

### Several FP-conjugated carrier proteins elicit HIV-1-neutralizing responses, with the highest overall response from FP-conjugated rTTHC

**Figure 2:**
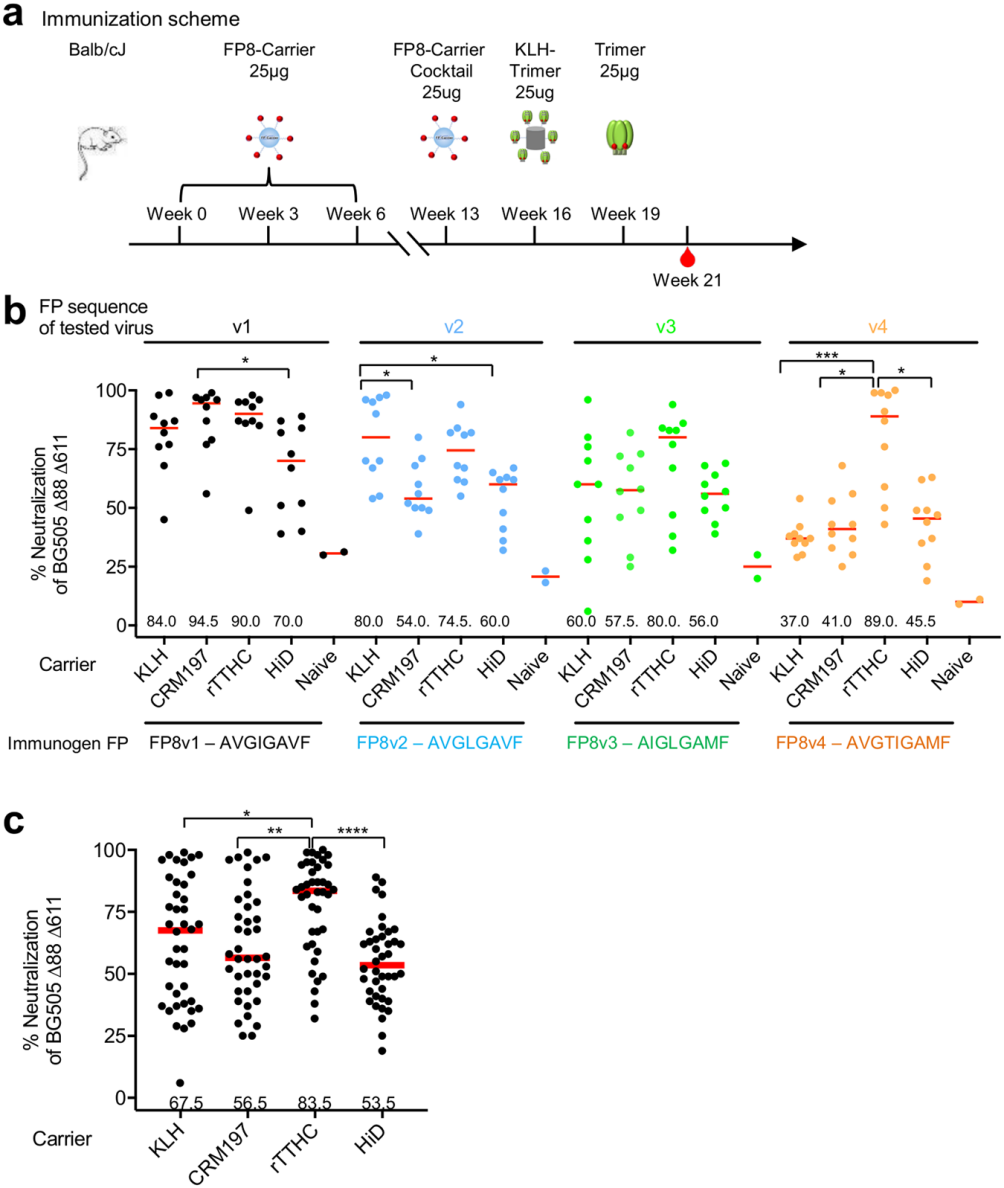
All tested FP-carrier conjugates elicited HIV-1 neutralizing responses, with FP-rTTHC eliciting the highest overall responses. (**a**) Immunization regimen. Groups of 10 mice were immunized 3 times with an FP-carrier immunogen with MBS linker and boosted with a cocktail of 4 FP-carrier conjugates, a KLH-BG505 Env trimer conjugate, and a BG505 Env trimer at the indicated times. For each group of mice, all immunogens contain the same FP sequence. Adjuplex was used as adjuvant. Serum samples were taken two weeks after the last immunization. (**b**) Virus neutralization assays. Week 21 immune sera at 1:20 dilution were assayed for neutralization of the BG505 Δ88Δ611 virus with indicated FP sequence. Naïve sera from pre-immunization were used as controls. Each dot represents the neutralization data from a single mouse. (**c**) Evaluation of the effect on elicited neutralization responses for each carrier protein. Data in (**b**) for each carrier protein were combined. Median values highlighted with red bars (values shown at the bottom); and *P* values calculated with Kruskal-Wallis test with a post hoc Dunn’s multiple-comparison test. *P < 0.05; **P < 0.01; ***P < 0.001; ****P < 0.0001.

To test the immunogenicity of the 16 FP8-carrier immunogens, we immunized 16 groups, each comprising 10 Balb/c mice, 3 times with one of the FP8-carrier conjugates. These three immunizations were followed by a boost with a carrier cocktail, comprising the four carrier proteins conjugated to the same FP sequence, and then by two additional boosts with BG505 Env trimer(BG505 DS-SOSIP) (Fig. 2a), as we previously observed boosting with Env trimer to substantially increase the titer of FP-directed neutralizing activity^2^.

With each of the trimer boosts, we matched the FP8 sequence on the BG505 Env trimer with the FP8 sequence that each group had been immunized by the FP8-conjugated carrier proteins. The first of the trimer boosts (week 16) involved the FP8-specific Env trimers conjugated to KLH and the second trimer boost (week 19) involved only Env trimers; we checked by negative-stain electron microscopy (EM) that the FP8v2, FP8v3 and FP8v4 modifications of the BG505 sequence did not alter the closed conformation of the Env trimer (Supplementary Fig. S2). Overall, with each group of immunized mice, all immunogens contained the same FP8 sequence.

We assessed week 21 immune sera for neutralization against the BG505 double glycan-deletion variant (Δ88 and Δ611), which is 10–100-fold more sensitive to neutralization by FP-directed antibodies than the wild-type BG505 virus^2,3^. For neutralization assessment of each FP variant group, we matched the N-terminal FP8 sequence of the double glycan-deleted viral variant with the FP8-sequence of the immunogen. Roughly half of the 16 FP8-carrier conjugates elicited average neutralizing responses of greater than 50% by week 21 (Fig. 2b). With all four FP8 sequences, conjugates made with the rTTHC carrier generally elicited higher neutralizing responses, with merged data from different FP8 immunogens revealing rTTHC to elicit significantly higher neutralization responses than the other three carrier proteins (Fig. 2c). Based on these results, we selected rTTHC as the carrier protein of choice for further study.

### Assessment of crosslinkers indicates Sulfo-SIAB to be preferred

**Figure 3:**
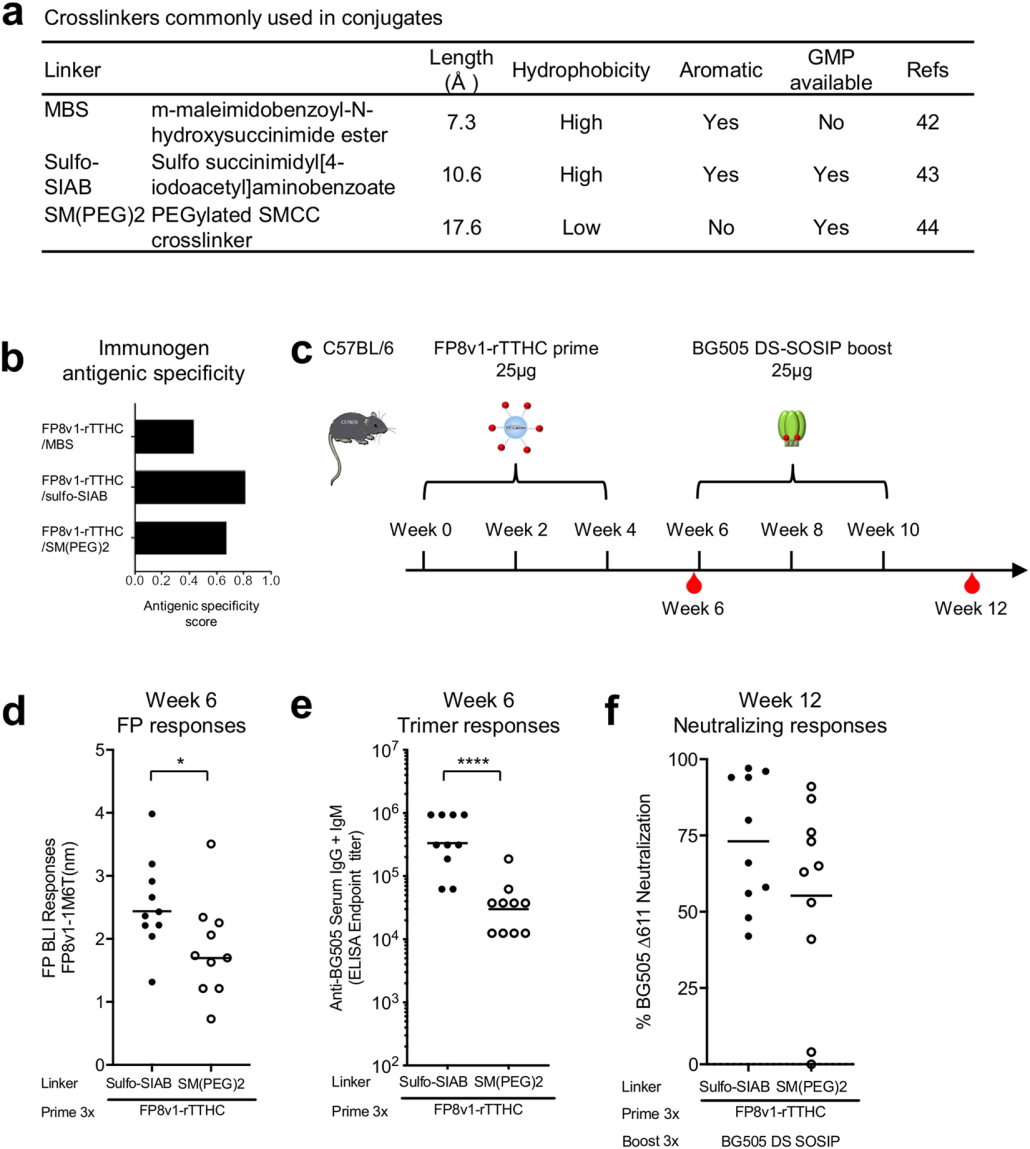
Assessment of linkers indicates Sulfo-SIAB to be preferred. (**a**) Properties of the commonly used crosslinkers in conjugate vaccines. (**b**) Immunogen antigenic specificity calculated by the published method^2^. **(c)** Immunization regimen. Mice were immunized three times with an FP8-rTTHC immunogen and boosted three times with BG505 Env trimer at the indicated times, with Adjuplex as adjuvant. Serum samples were taken two weeks after the last FP8-rTTHC immunization and the last BG505 trimer immunization. (**d**) Anti-FP response before the BG505 Env trimer immunization, measured by BLI responses to FP8v1-1M6T scaffold. (**e**) Anti-BG505 trimer response before the BG505 Env trimer immunization. (**f**) Virus neutralization assays with the immune sera at week 12. Sera at 1:50 dilution were used to neutralize the BG505 Δ611 virus. Median values indicated by horizontal black lines. *P* values calculated with Mann-Whitney two-tailed t test: *P < 0.05; **P < 0.01; ***P < 0.001; ****P < 0.0001.

Having selected rTTHC as the carrier of choice, we set out to determine a suitable reagent to link peptide and rTTHC (Fig. 3). Crosslinkers are a crucial component of conjugate vaccines^39–41^, and several heterobifunctional crosslinkers containing suitable amine- and sulfhydryl-reactive functional groups have been used in peptide-carrier-based vaccines. Previously^1,2,12^ as well as with the initial 16 FP-carrier conjugates tested, we used MBS^42^, a relatively hydrophobic linker of ~8 Å, to couple FP with carrier protein. Although one of the most widely used heterobifunctional linkers, MBS can cause skin and respiratory irritation and has been deemed hazardous for clinical assessment. We therefore evaluated two water soluble heterobifunctional linkers, which have been deemed GMP-suitable. These crosslinkers are sulfo succinimidyl[4-iodoacetyl]aminobenzoate and PEGylated SMCC^43,44^ (hereafter referred to as Sulfo-SIAB and SM(PEG)2) (Fig. 3a and Supplementary Fig. S3f). Although Sulfo-SIAB contains a light sensitive sulfhydryl-reactive function group, it is more stable in solution than maleimide-based crosslinkers like MBS, and equally hydrophobic. By contrast, SM(PEG)2 contains a hydrophilic spacer, and conjugates made with this linker generally have increased solubility.

We prepared FP8v1-rTTHC conjugates with Sulfo-SIAB or SM(PEG)2 linkers and assessed their antigenicity relative to a panel of FP-directed neutralizing antibodies (Fig. 3b) and their immunogenicity in mice (Fig. 3c). BLI-assessed antigenicity with FP-directed broadly neutralizing antibodies showed immunogen antigenic specificity for MBS-linked FP conjugates to be lower than that for SM(PEG)2- or Sulfo-SIAB-linked FP-conjugates, with Sulfo-SIAB-linked FP-conjugates exhibiting the highest level of immunogen antigenic specificity (Fig. 3b).

To evaluate immunogenicity, C57BL/6 mice (2 groups of 10 mice) were immunized in two-week intervals three times with FP8v1-rTTHC with either Sulfo-SIAB or SM(PEG)2 linker and then boosted 3 times with the BG505 Env trimer (Fig. 3c). Anti-FP and anti-trimer immune responses were measured before the Env trimer immunization at week 6. The SIAB group elicited higher immune responses against both FP and BG505 Env trimer (Fig. 3d,e). After additional three boosts with BG505 Env trimer, the neutralization response against the BG505 Δ611 glycan variant trended higher for the Sulfo-SIAB group than that for the SM(PEG)2 group (Fig. 3). These results suggested Sulfo-SIAB to be a preferred linker over SM(PEG)2 for FP-conjugate vaccine immunogens.

### Immunization with multiple carriers – either as cocktail mixtures or sequentially – does not induce improved HIV-1 neutralizing responses

**Figure 4:**
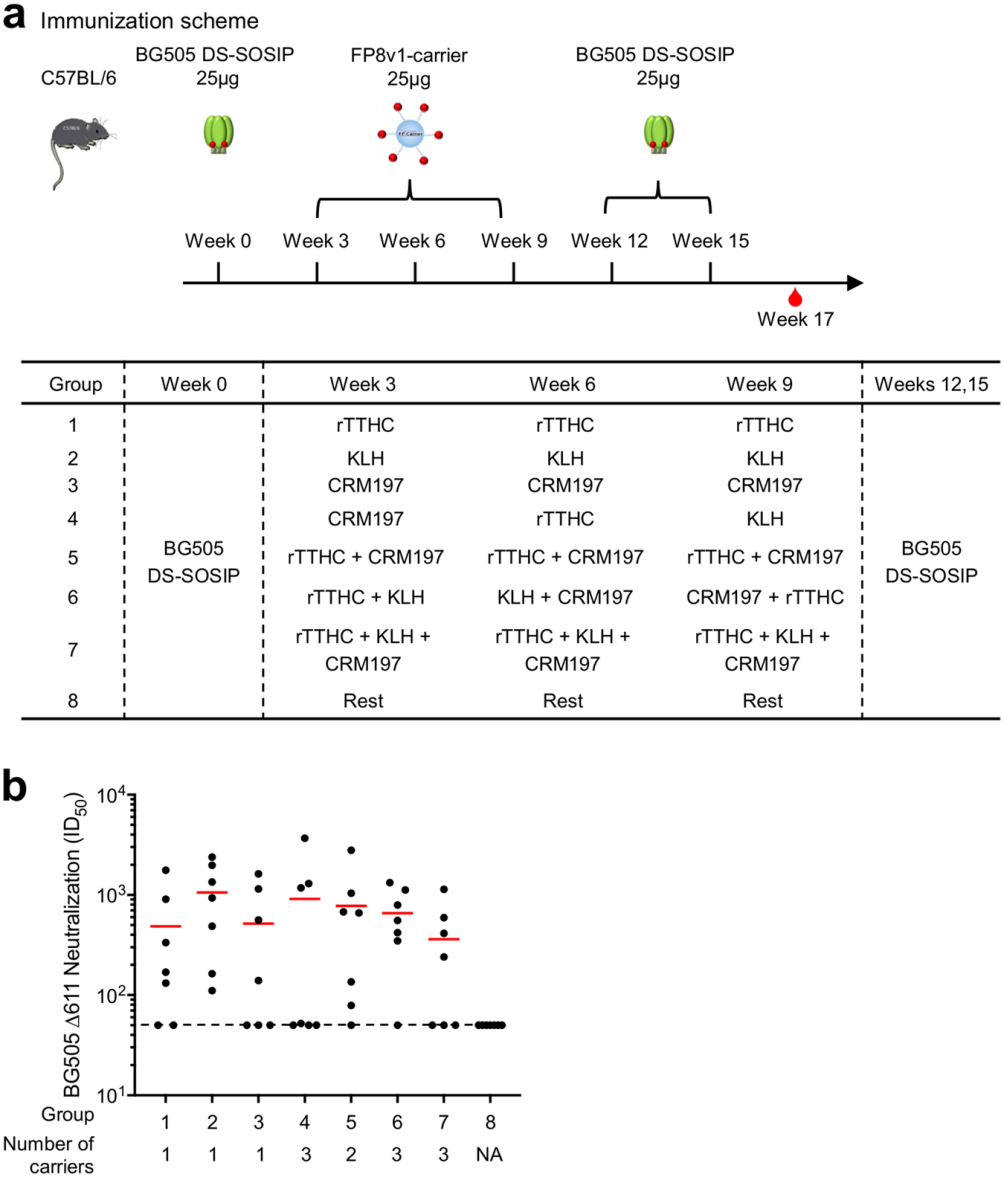
Immunization with multiple carriers (either cocktail or sequential) does not induce improved HIV-neutralizing responses. (**a**) Immunization regimen. Mice were immunized once with BG505 Env trimer, three times with diverse FP8v1-carrier immunogens, and then twice with BG505 Env trimer at the indicated times. (**b**) Virus neutralization assays. Week 17 immune sera at 1:50 dilution were used to neutralize the BG505 Δ611 virus, dotted line is the threshold of assay. Mann Whitney test was used to compare the statistical difference between each group and group 8, which did not have any FP-carrier immunization and did not neutralize BG505 Δ611. Mean values highlighted as red bars.

In addition to using carrier proteins individually, combinations of protein carriers – either as cocktail mixtures or as sequential immunogens – have the potential to increase immunogenicity^45^. To evaluate possible synergy between the various carriers, we assessed vaccine regimens comprising a BG505 Env trimer prime at week 0, various peptide-carrier immunogen boosts at weeks 3, 6 and 9, and then two additional BG505 Env trimer boosts at weeks 12 and 15 (Fig. 4a). For the various peptide-carrier conjugate boosts, we coupled FP8v1 to carriers with the Sulfo-SIAB linker and tested single carriers (groups 1–3), single carriers altered sequentially (group 4), cocktails of two carriers either with rTTHC and CRM197 (group 5) or as sequentially alternating combination of two carriers (group 6), cocktails of three carriers (group 7) as well as a no carrier control group (group 8). The total amount of FP8v1-carrier conjugates for each immunization in each of the groups was the same (25 ug). For immune readout, we assessed neutralization at week 17 against the mutant BG505 Δ611 virus, which had the FP8v1 native sequence and like the Δ88Δ611 double mutant was especially sensitive to FP-directed neutralizing responses (Fig. 4b). Notably, we observed titers for groups 1–7 to be statistically indistinguishable from each other (Fig. 4b). Thus, we did not observe different combinations of carrier immunogens – either in cocktail mixtures or altered sequentially – to significantly impact the elicited neutralization titers.

### Different ratios of peptide-carrier, adjuvanted with Adjuplex or with Alum, can induce desired FP-directed neutralizing responses

**Figure 5:**
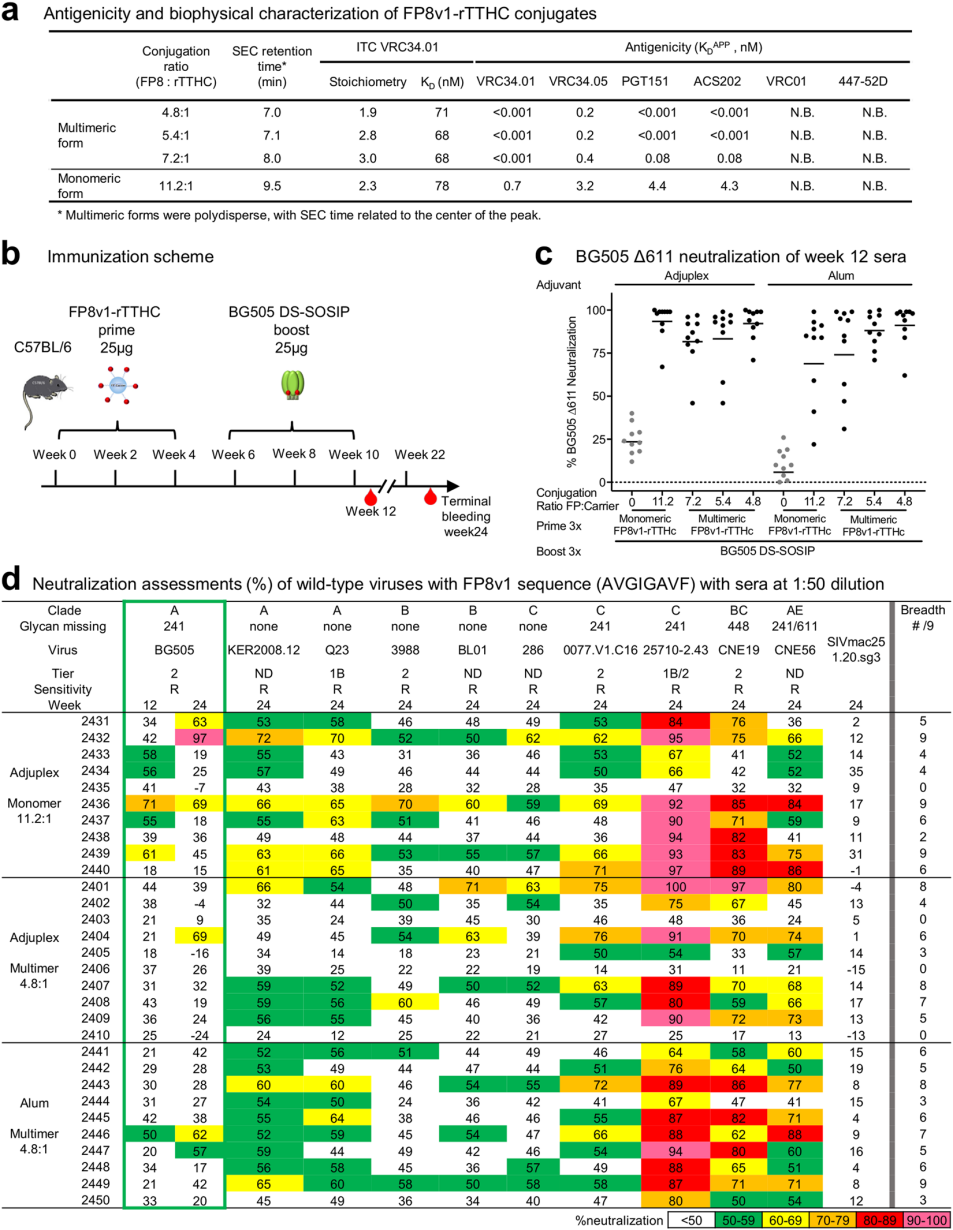
Cross-clade neutralizing responses can be induced by different ratios of peptide-carrier, adjuvanted with either Adjuplex or Alum. (**a**) Antigenicity and biophysical characterization of various forms of FP8v1-rTTHC conjugates. Size exclusion chromatography were performed with Superose 6 column using PBS buffer. Stoichiometry and K_D_ were measured with ITC by titrating antibody VRC34.01 Fab into FP-rTTHC conjugate at 25 °C. Antigenicity K_D_^APP^ values were measured with BLI. N.B., no detectable binding, K_D_ > 1 µM. (**b**) Immunization regimen. Mice (10 per group) were immunized three times with an FP8v1-rTTHC and boosted three times with BG505 at the indicated times, with either Adjuplex or Alum as adjuvant. (**c**) Serum neutralization of BG505 Δ611. Week 12 sera were diluted 1:50. Mean values highlighted as black bars. (**d**) Serum neutralization of autologous BG505 (green box) and 9 FP-matching heterologous wild-type strains. Sera were diluted 50 fold for the neutralization assay. Number of heterologous strains being neutralized at least 50% for each mouse serum is listed on the right column.

Having selected rTTHC and Sulfo-SIAB as carrier and linker, we assessed immunogenically additional variants of FP8v1-rTTHC as monomeric as well as multimeric forms of the conjugates, with varying peptide conjugation ratio ranging from 4.8 to 11.2 FPs attached to each rTTHC (Fig. 5a and Supplementary Figs. S4 and S5 and Table S1). The monomeric form of FP8v1-rTTHC had 11.2 copies of FP8 conjugated to each rTTHC, as determined by amino acid analysis, and on the multimeric forms of FP8v1-rTTHC, amino acid analysis indicate there were FP8 to rTTHC ratios of 4.8~7.2:1. Isothermal titration calorimetry (ITC) revealed N123-VRC34.01 K_D_s of 70–80 nM for all forms of FP8v1-rTTHC and stoichiometries ranging between 1.9 and 3.0 for the number of antigenic-binding fragments (Fabs) of N123-VRC34.01 that could bind each FP8v1-rTTHC monomer (Fig. 5a and Supplementary Fig. S4c), suggesting that not all FP8 peptides conjugated to rTTHC were accessible for VRC34.01 binding presumably due to steric hindrance between Fabs binding to proximal FPs. BLI measurements with N123-VRC34.01 IgG indicated an apparent affinity of tighter than 1 pM, reflecting increased affinity related to avidity from each arm of the antibody binding to a separate FP. We also observed high specificity, with only FP-directed antibodies binding to the FP8v1-rTTHC conjugates (Fig. 5a and Supplementary Fig. S5).

We immunized mice (n = 10/group) with these 4 FP8v1-rTTHC immunogens or rTTHC alone 3 times at weeks 0, 2, and 4, and then boosted 3 times with BG505 Env trimer at weeks 6, 8, and 10, using either Alum or Adjuplex as an adjuvant (Fig. 5b and Supplementary Fig. S5). We monitored FP-directed immune responses by measuring serum binding to an FP-scaffold protein (FP8v1_1M6T) and to Env trimer (BG505 DS-SOSIP) by ELISA (Supplementary Fig. S5). While Adjuplex and Alum groups exhibited statistically similar ELISA titers against BG505 trimer after the third FP8v1-rTTHC priming (Supplementary Fig. S5b), we observed that (i) the Adjuplex groups elicited ~2 fold higher anti-FP immune response than the Alum groups, (ii) the difference in anti-FP response between groups using the two different adjuvants to be reduced with multivalent immunogens, and (iii) anti-FP responses of the Adjuplex groups plateaued after the second FP8v1-rTTHC immunization, whereas in the Alum groups the anti-FP responses continue to increase, with a 50–100% increase after the second BG505 Env trimer boost (Supplementary Fig S5c,d). Neutralizing responses against BG505 Δ611 were similar between the Adjuplex groups and Alum groups for the multimeric FP8v1-rTTHC immunogens, whereas for the monomeric form of FP8v1-rTTHC, the Adjuplex group exhibited higher neutralizing responses than the Alum group (P < 0.01) (Fig. 5c).

To analyze the neutralization breadth of the immune sera, we selected 3 groups: two Adjuplex groups immunized with FP8v1-rTTHC, the monomer with 11.2:1 ratio and the multimer with 4.8:1 ratio, and one Alum group immunized with FP8v1-rTTHC multimer with 4.8:1 ratio. Week 24 immune sera, after three Env trimer boosts, were assessed for neutralization on a panel of 10 wild-type strains, selected to encompass divergent HIV-1 clades (Fig. 5d). Homologous neutralization against BG505 was observed only sporadically (Fig. 5d), as was the neutralization against HIV-1 strains with non-matching FP8 sequences (Supplementary Fig. S6). An exception was the strain TH023.6 (Supplementary Fig. S6), which has FP Thai sequence^1^ with the first six residues identical to those in FPv1, suggesting that the N-terminal six residues are likely to play a more important role for FP-directed antibody recognition, especially for the recognition of this TH023.6 strain. Heterologous neutralization of viruses with matching FP8 sequences, however, was much stronger, with the majority of mice in all three groups neutralizing at least five out of nine heterologous strains (Fig. 5d). All three groups exhibited a similar level of neutralization against heterologous strains with no statistically significant difference, suggesting that, in the mouse model, both Alum and Adjuplex were suitable as adjuvants and that multimerization of conjugate immunogens or variation of FP to carrier ratios did not substantially impact the elicitation of neutralizing responses.

## Discussion

With immunization involving FP linked to KLH eliciting cross-clade neutralizing responses of surprising breadth against HIV-1 in standard vaccine-test animals including mice, guinea pigs and rhesus macaques^1,2,12^, it is becoming ever more critical to test FP-conjugated carriers clinically, to determine if similar broad responses against FP can be elicited by vaccination of humans. Unfortunately, the research grade FP-carrier and conjugating linker, which were tested in initial vaccine-test animal experiments, are not suitable for testing in humans. Here we identify rTTHC as a suitable carrier protein and Sulfo-SIAB as a suitable crosslinker for clinical development of FP-carrier conjugate immunogens. We further assessed the immunogenicity of a number of FP8v1-rTTHC variants and found these to elicit cross-reactive neutralizing responses, which were tolerant to variation in ratio of FP linked to each rTTHC and to the type of adjuvant used (Fig. 5). Such tolerance suggests the elicited broad response to be primarily dependent on use of FP and carrier and to be robust to many of the specific characteristics of peptide, carrier, and their conjugation.

In general, immunization with peptides is a well-established technology^46–48^. In many cases, peptide immunization fails to elicit high titer-neutralizing antibody responses, because the conformationally unstructured peptide immunogens often elicit antibodies that cannot bind natively folded protein antigens^49,50^. FP in the context of HIV Env trimer, however, is conformationally diverse, as observed in crystal and cryo-EM structures of Env trimers^5,6^, by molecular dynamics simulations of Env trimers^3^, and by diversity in the conformation of FP recognized by cross-clade neutralizing antibodies^2,51^. The inherent structural diversity of FP on the prefusion-closed Env trimer thus appears to mirror the conformational diversity of FP coupled to carrier protein, with antibodies elicited by conformationally diverse FP immunogens still able to recognize and neutralize FP in the Env trimer context. We note that the ability to elicit broad HIV-1 neutralizing responses with an established vaccine technology has multiple advantages, including the prior development and clinical assessment of multiple carrier proteins.

Several toxoids, including those derived from diphtheria and tetanus toxins, are components of carrier protein vaccines^16–19^, and recombinant versions of each have been developed. rTTHC, a well-characterized fragment of TT that is produced by recombinant DNA technology and can be manufactured as a GMP product. rTTHC retains the most potent T helper epitopes of TT and could induce higher immune response than the assembled KLH nanoparticle, which is ~150-fold larger than rTTHC in molecular weight. As combinations of carriers did not improve immunogenicity (Fig. 4), rTTHC appears to contain sufficient T helper epitopes that could not be substantially improved by either cocktail or sequential addition of other carrier proteins.

While FP-carrier protein priming is important for initiating FP-directed responses^12^, Env trimer boosts are also important to mature the responses into potent broad neutralization^1,2,12^. Furthermore, our results suggest that compared to Adjuplex, use of Alum as an adjuvant resulted in slower development of anti-FP responses during the FP8v1-rTTHC priming stage, though these responses continued to develop upon trimer boosting, ultimately yielding similar responses as induced by Adjuplex at the end of the study. Our findings highlight that using multiple FP primes and multiple trimer boosts can eventually achieve the desirable immune response even with detectable different response after the FP primes using different adjuvants.

Although there is no substantial impact on the elicitation of immune responses among the conjugates with variable conjugation ratios, our data suggest that the group immunized with multivalent immunogens with larger size has less deviation (Fig. 5c, Supplemental Figs. S4a,b and S5). Interestingly, the anti-FP response appears to reach saturation earlier when using the heavily self cross-linked immunogen (Supplemental Fig. S5). This suggests that design of nano-particle based carrier immunogens are desirable for the peptide-based conjugation immunogens.

Although further development of FP8-rTTHC will require clinical assessment, it may be possible to optimize – in parallel – neutralizing responses of FP8-rTTHC in vaccine-test species. In this regard, it seems likely that the incorporation of toll-like receptor ligands or dendritic cell targeting will improve immunogenicity. Because FP immunogens can reproducibly elicit cross-clade neutralizing antibodies against a defined site of vulnerability, experiments to further optimize FP immunogens and immunization regimens and to confirm the elicitation of broad HIV-1 neutralizing responses in humans are a priority that the definition of rTTHC carrier and Sulfo-SIAB linker as appropriate GMP reagents has moved one step closer to achieving.

## Materials and Methods

### Ethics statement

All mice experiments were reviewed and approved in protocol VRC-16-688 by the Animal Care and Use Committee of the Vaccine Research Center, National Institutes of Allergy and Infectious Diseases (NIAID), National Institutes of Health (NIH) and all animals were housed and cared for in accordance with local state, federal and institute policies in an American Association for Accreditation of Laboratory Animal Care-accredited facility with stringent standard operating procedures and compliant with *U.S. Animal Welfare Act (AWA) and Regulations*, the *Public Health Service (PHS) Policy on Humane Care and Use of Laboratory Animals*, the *Guide for the Care and Use of Laboratory Animals* and all applicable NIH Policies on *in vivo* research. Animal procedures were conducted in strict accordance with all relevant federal and National Institutes of Health guidelines and regulations.

### Animal protocols and immunization

Mice were housed and cared for in accordance with local, state, federal, and institute policies in an American Association for Accreditation of Laboratory Animal Care-accredited facility at Vaccine Research Center, NIAID, NIH.

Female C57BL/6 or Balb/cJ mice with body weights of over 17 g were obtained from Jackson Laboratory (Wilmington, MA) for immunization studies. For each immunization, 25 μg of immunogen formulated with either 20% of Adjuplex (Empirion LLC, Columbus, OH or Adjuplex equivalent formulated based on US Patent 6,676,958 B2) or 250 μg of Aluminum hydroxide gel (Brenntag, PA) in the final volume of 100 μL, were injected intramuscularly to the caudle thigh of the two hind legs. Blood was collected for serological analysis.

### Cell lines

Expi293F cells were from ThermoFisher Scientific Inc (Invitrogen, cat# A14528; RRID: CVCL_D615). TZM-bl cells were from NIH AIDS Reagent Program (www.aidsreagent.org, cat# 8129). HEK 293T/17 cells were from ATCC (cat# CRL-11268).

### Fusion peptide immunogens

Protein KLH were obtained from Thermo Fisher Scientific Inc. CRM197 were obtained from Fina BioSolutions. rTTHC were obtained from Fina BioSolutions, LLC or produced in VRC Production Program. The gene of HiD were synthesized and subcloned to the vector of pGEX-4T-1(GenScript, Piscataway, NJ) and the protein were expressed in the *E. coli* BL21 (DE3). HIV-1 fusion peptide (FP8v1: AVGIGAVFC, FP8v2: AVGLGAVFC, FP8v3: AIGLGAMFC, FP8v4: AVGTIGAMFC) were synthesized (GenScript, Piscataway, NJ) with a free amine at the N terminus and an extra cysteine residue at the C terminus. KLH, CRM197, rTTHC and HiD conjugates were prepared via in two steps. The first is the activation of carrier protein using cross linker (MBS, m-maleimidobenzoyl-N-hydroxysuccinimide ester; Sulfo-SIAB, SM(PEG)2 (PEGylated SMCC crosslinker), this was followed by coupling of terminal thiol to the maleimide or iodoacetyl group of the activated carrier proteins. The antigenicity of the conjugates was confirmed by binding of fusion peptide specific antibodies VRC34.01, VRC34.05, PGT151 and ACS202. For rTTHC, the conjugation ratio of FP to carrier protein was defined by amino acid analysis. It was calculated based on the changes in the amino acid composition of amino acids present in FP8 versus the composition of amino acids not present in FP8. The ratio of FP8 to rTTHC in the conjugate was determined by using quantification of rTTHC by amino acids, which were not in FP8v1 and passed quality control (Arg, Leu, Lys and Pro; we excluded the outlier and averaged quantification based on the remaining three amino acids) and quantification of FP8 with the five amino acid in the FP8v1 sequence (Ala, Gly, Val, Ile and Phe), excluding the upper and lower outliers, and averaged quantification based on the remaining three amino acids (Supplementary Table S1).

### HIV-1 envelope trimer

HIV BG505 Env trimers (BG505.DS-SOSIP_v1, v2, v3 and v4) produced in transiently transfected 293F cells has described previously^52^. Briefly, 0.75 mg of mixture of the plasmid encoding the trimer and the plasmid encoding human furin at 4:1 ratio were used to transfected 293 F cells using 293Fectin (Thermo Scientific) or Turbo293 transfection reagent (Speed BioSystems). Cells were incubated in shaker at 120 rpm, 37 °C, 9% CO_2_. On the next day of transfection, 80 ml HyClone SFM4HEK293 medium and 20 ml FreeStyle 293 Expression Medium were added to each liter of cells. The Env trimer was purified from the day-7 supernatant using 2G12 or VRC01 antibody affinity chromatography, followed by size exclusion column on a Sephadex200 16/60HL, finally the V3 epitope exposed species were removed through the 447-52D antibody affinity column. Purified envelope trimers were analyzed by the negative-stain electron microscopy^2^. BG505 DS-SOSIP trimers were also expressed from a CHO-DG44 stable cell line and purified using a series of non-affinity chromatography steps. The antigenic profile of 293 F and CHO-expressed Env trimers were similar, as assessed by Meso Scale Discovery platform.

The KLH-trimer conjugates were prepared using maleimide-and-hydrazide crosslinker N-κ-maleimidoundecanoic acid hydrazide (KMUH). Briefly, 1 mg BG505 DS-SOSIP were incubated with equal molar of sodium meta-periodate on ice for 30 min; and KLH were treated with succinimidyl 3-(2-pyridyldithio)propionate (SPDP) followed by the activation with KMUH. Finally, KLH and trimer were mixed at 4 degree overnight and conjugates were purified by size exclusion chromatography.

### Negative-stain electron microscopy

Env trimers were diluted to 0.02 mg/ml with a buffer containing 10 mM HEPES, pH 7, and 150 mM NaCl. Diluted samples were applied to a freshly glow-discharged carbon-coated copper grid for approximately 15 s. The grid was rinsed with the above buffer, and adsorbed protein molecules were negatively stained with 0.7% uranyl formate. Datasets were collected using SerialEM^53^ on an FEI Tecnai T20 microscope equipped with an Eagle CCD camera at a nominal magnification of 100,000 (pixel size: 0.22 nm). Particles were selected from micrographs automatically using in-house developed software (YT, unpublished). Reference-free 2D classification was performed using EMAN2 software package^54^.

### Antigenic characterization

A FortéBio Octet HTX instrument was used to measure binding kinetics of conjugates to antibodies. All assays were performed with phosphate-buffered saline (PBS) with 1% bovine serum albumin (BSA) to minimize nonspecific interactions at 30°C. AHC sensor tips (FortéBio) were used to capture antibodies for 300 s. Biosensor tips were then equilibrated for 60 s in buffer prior to measuring association with immunogens for 300 s followed by 300 s dissociation. Experimental data were analyzed using Global fitting with 1:1 model binding using Octet software, version 9.0.

### ITC studies

ITC was carried out at 25 °C using a VP-ITC microcalorimeter from MicroCal Malvern Instruments (Northampton, MA, USA). All reagents were dialyzed to the PBS buffer and thoroughly degassed prior to the titration. For all the experiments, VRC34.01 Fab was added stepwise in 7–10 uL aliquots to the calorimetric cell (v ~ 1.4 mL) containing the FP conjugates at 0.25–0.50 µM. The concentration of VRC34.01 Fab in the syringe was 20–45 µM.

### Anti-trimer (BG505 DS-SOSIP) Enzyme-Linked Immunosorbent Assay (ELISA)

Anti-trimer responses in the immunized mice were analyzed using an in-house developed lectin capture ELISA. The ELISA methodology has been described previously^1^. Briefly, the 96 well plates were coated with snowdrop lectin to capture the glycosylated trimer. Serially diluted mouse sera were added and incubated for 1 hour at room temperature followed by the goat anti-mouse antibody incubation. Plates were read at 450 nm after developed with tetramethylbenzidine (TMB) substrate for 10 min before the reaction was stopped with1 N sulfuric acid. The optical densities (OD) were analyzed following subtraction of the nonspecific horseradish peroxidase background activity. The endpoint titer was defined as the reciprocal of the greatest dilution with an OD value above 0.1 (2 times average raw plate background).

### Sera antigenic analysis

Mouse sera from a subset of immunization groups were assessed for binding to scaffold protein FP8-1M6T with a FortéBio Octet HTX instrument. Sera were diluted 1:100 in 1% BSA/PBS. NTA sensor tips obtained from FortéBio were equilibrated in 1% BSA PBS before the assays. The NTA biosensors were loaded with FP8v1-1M6T at 20 μg/ml in 1% BSA/PBS for 300 s followed by equilibrated in buffer for 60 s. The sera responses were measured by the association step for 300 s in sera followed by a dissociation step for additional 60 s. The naive prebleed sera response for each group of immunogens was used as a reference. All experiments were performed in duplicate, and the average of the two data sets was analyzed with Octet and GraphPad Prism 8 software.

### Neutralization assays

Neutralization was measured using single-round-of-infection HIV-1 Env-pseudoviruses and TZM-bl target cells, as described previously^1^. We used either Δ88Δ611 or Δ611 mutant of BG505 to assess FP-directed responses. Both mutants are sensitive tools for assessing FP-directed responses, with the double glycan-deletion mutant being more sensitive. However, some FP-directed antibodies require glycan N88 for recognition of HIV Env. Neutralization curves were fit by nonlinear regression using a 5-parameter hill slope equation. For sera, the 50% and 80% inhibitory dilutions (ID50 and ID80) were reported as the reciprocal of the dilutions required to inhibit infection by 50% and 80%, respectively. Single-point assays were performed in duplicate at a dilution of 50, and data reported as % neutralization.

## Supplementary information

Supplementary information.

## Data Availability

All relevant data are within the paper and its Supporting Information files.
