# Supplementary material for: Preclinical Development of a Fusion Peptide Conjugate as an HIV Vaccine Immunogen
Source: Sci Rep. 2020 Feb 20;10:3032. doi: 10.1038/s41598-020-59711-y (PMC7033230; doi:10.1038/s41598-020-59711-y)
Supplement: Supplementary file 1 — Supplementary information. [file 41598_2020_59711_MOESM1_ESM.pdf]

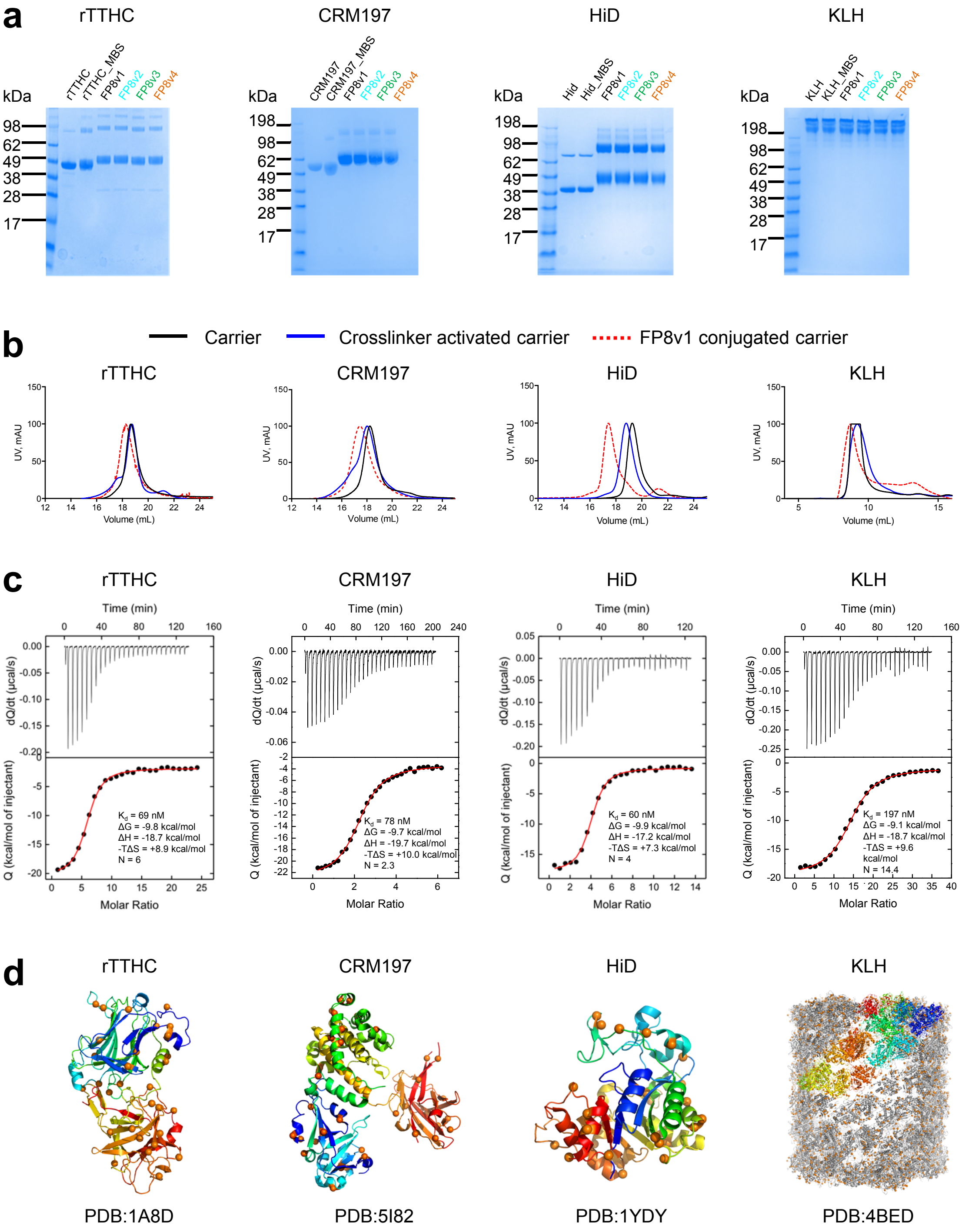

Figure S1. Biophysical characterization of the 16 FP8-carrier conjugates. (**a**) SDS-PAGE. For each carrier protein, the carrier protein, crosslinker activated carrier, and its FP conjugates are shown on a gel. (**b**) Analytical SEC of the carrier protein, crosslinker activated form, and FP8v1 conjugate. (**c**) ITC characterization of FP8v1-carrier conjugates by titration with VRC34.01 Fab. (**d**) Structures of rTTHC, CRM197 and KLH and a homology model of HiD based on PDB 1YDY. The lysine residues on the structures of rTTHC, CRM197, and KLH are highlighted with orange spheres. *Continued on next page*

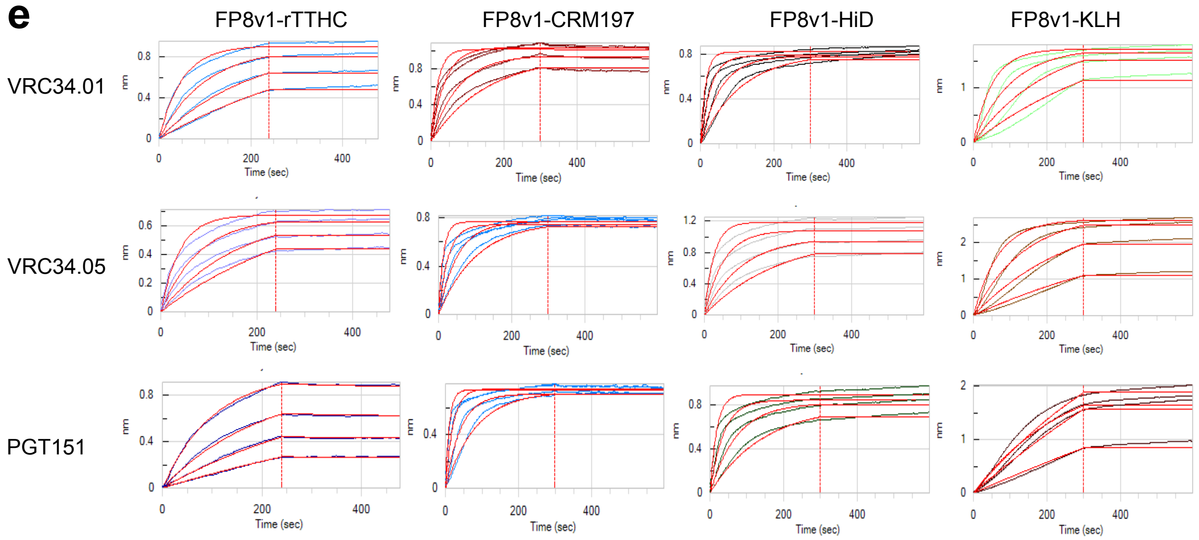

	VRC34.01			VRC34.5			PGT151		
	KD (nM)	kon(1/Ms)	kdis(1/s)	KD (nM)	kon(1/Ms)	kdis(1/s)	KD (nM)	kon(1/Ms)	kdis(1/s)
**FP8v1-rTTHC**	<0.001 (±0.01)	3.20E+05	<1.0E-07	0.3 (± 0.09)	2.02E+05	7.05E-05	<0.001 (± 0.06)	1.91E+05	<1.0E-07
**FP8v1-CRM197**	<0.001 (± 0.01)	4.82E+05	<1.0E-07	0.06 (± 0.01)	2.66E+05	1.77E-05	<0.001 (± 0.005)	3.87E+05	<1.0E-07
**FP8v1-HiD**	<0.001 (± 0.078)	3.60E+05	<1.0E-07	<0.001 (± 0.065)	1.96E+05	<1.0E-07	<0.001 (± 0.121)	2.77E+05	<1.0E-07
**FP8v1-KLH**	<0.001 (± 0.005)	1.81E+06	<1.0E-07	<0.001 (± 0.006)	7.87E+05	<1.0E-07	<0.001 (± 0.034)	7.80E+05	<1.0E-07

Figure S1. Biophysical characterization of the 16 FP8-carrier conjugates. (**e**) Antigenicity assessment of FP immunogens by BLI method with Octet.

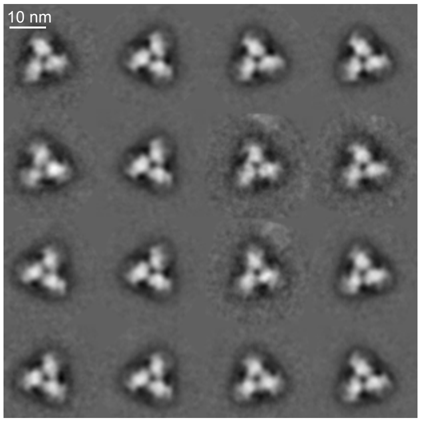

BG505 DS-SOSIP FPv1

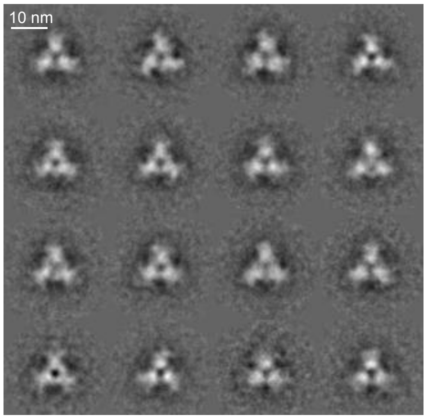

BG505 DS-SOSIP with FPv2

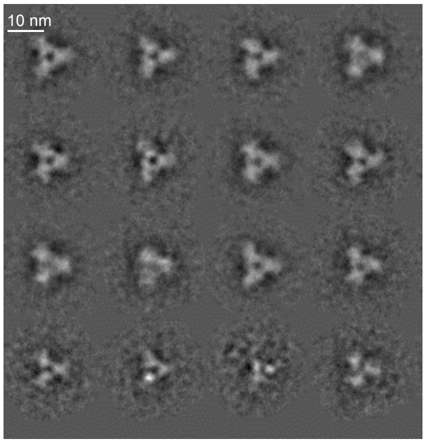

BG505 DS-SOSIP with FPv3

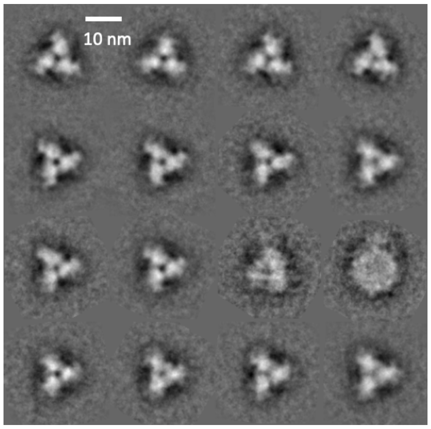

BG505 DS-SOSIP with FPv4

Figure S2. Negative-stain EM images of BG505 Env trimer and its mutants with an altered FP sequence confirm prefusion-closed conformation. Representative 2D class averages illustrated symmetrical propeller-like prefusion-closed HIV-1 Env trimer.

a

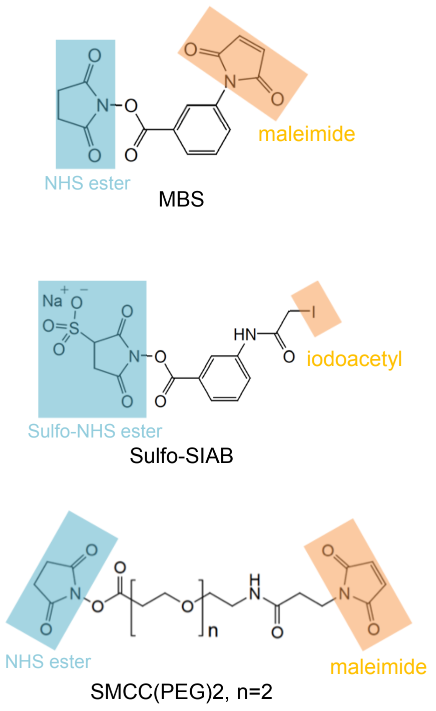

b Antigenicity of FP8v1-rTTHC conjugates with 3 different crosslinkers

Conjugates	Antigenicity (K_D^{APP} , nM)					
	VRC34.01	VRC34.05	PGT151	ACS202	VRC01	447-52D
FP8_v1-rTTHC/MBS	0.3	1.3	2.3	38.2	N.B.	N.B.
FP8_v1-rTTHC/Sulfo-SIAB	<0.001	<0.001	<0.001	31.0	N.B.	N.B.
FP8_v1-rTTHC/SM(PEG2)	<0.001	0.07	0.03	35.1	N.B.	N.B.

Figure S3. (a) Chemical structure of crosslinkers used for the conjugation. Functional groups reactive to amino or sulfhydryl are highlighted in light blue or orange. (**b**) Antigenicity of FP8v1-rTTHC conjugates with 3 different crosslinkers.

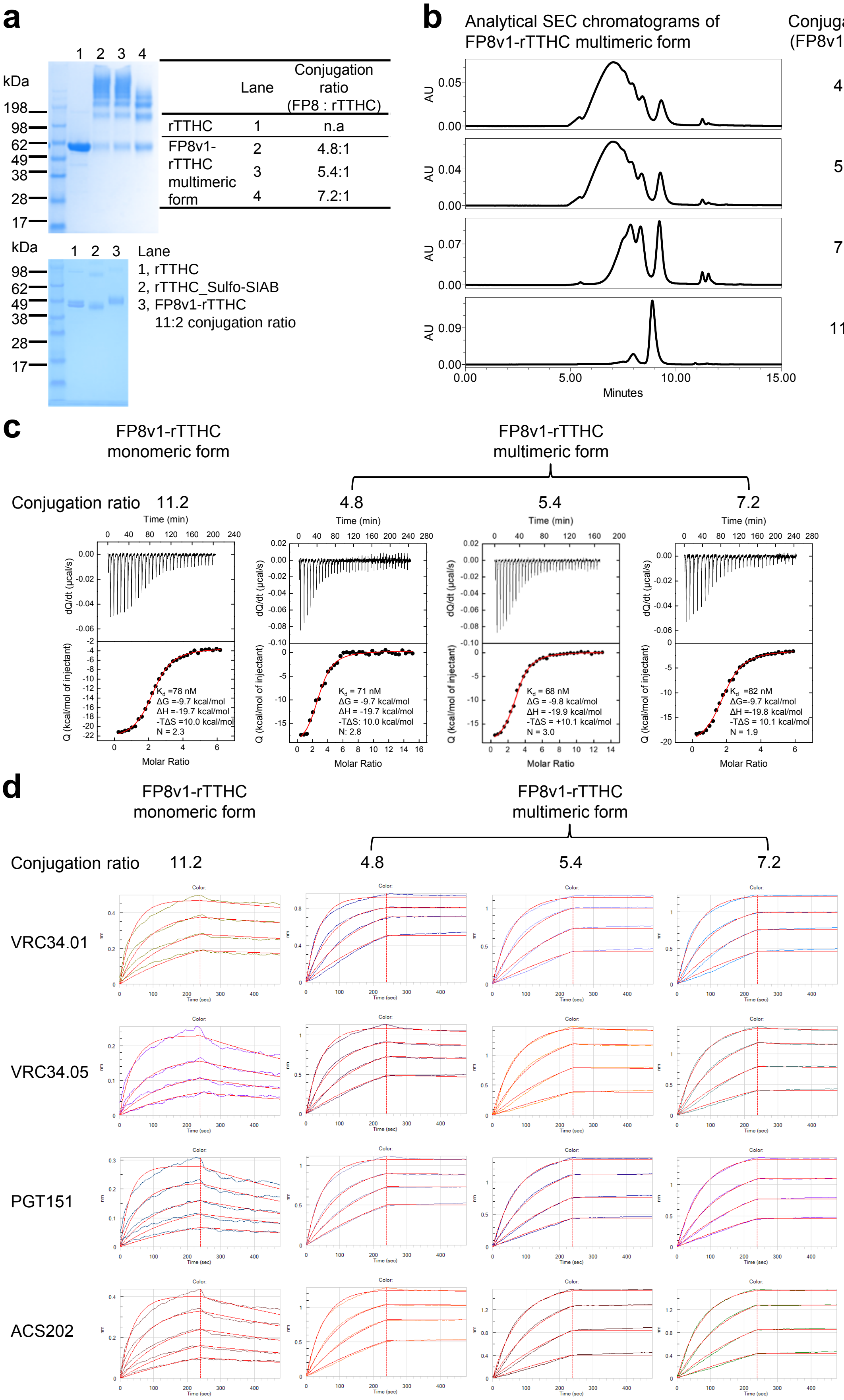

Figure S4. Physical characterization of rTTHC-conjugate products. (**a**) SDS-PAGE of the purified conjugates. (**b**) Analytical SEC of FP8v1-rTTHC conjugates with different ratio of FP8 loading. SEC was performed using an Agilent AdvanceBio SEC 300Å column in 2xPBS at a flow rate of 0.3 mL/min. (**c**) Stoichiometry and K_D were measured with ITC by titrating antibody VRC34.01 Fab into FPv1-rTTHC conjugates. (**d**) Antigenicity characterization of FP immunogens by BLI method with Octet.

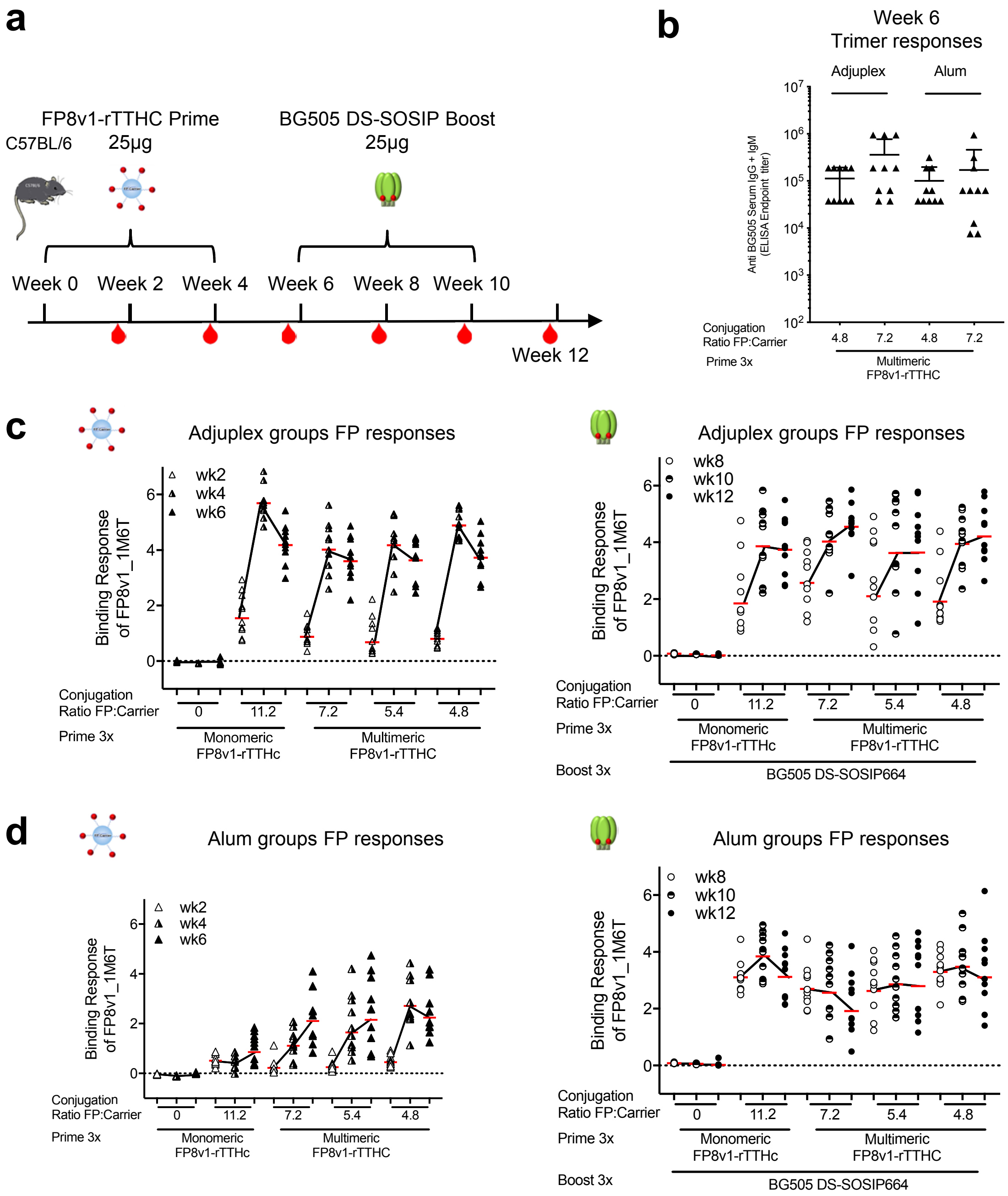

Figure S5. Different ratios of peptide-carrier, adjuvanted with Adjuplex or Alum, can induce the desired FP-directed neutralizing response. (**a**) Immunization regimen. Mice were immunized three times with an FP8-rTTHC immunogen, boosted three times with BG505 at the indicated times. Serum samples were taken two weeks after each immunization. (**b**) Anti-BG505 DS-SOSIP response before the trimer boost. (**c, d**) Antigenic analysis of sera from immunized mice adjuvanted with Adjuplex and Alum, sera from mice immunized with FP8-rTTHC conjugates with different coupling ratio were assessed for binding to FP scaffold protein(1M6T-FP8v1).

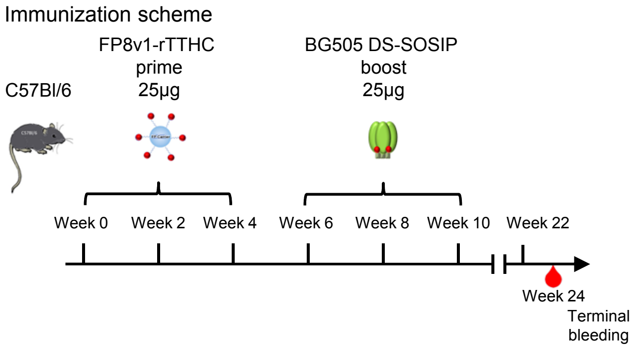

	Virus	BI369.9A	0815.V3.C13	T278-50	6644.V2.C33	6405.v4.c34	A03349M1	ADA.DG.SG3	C4118.09	TH023.6	SVA.MLV	SIVmac251 .30.SG3
		**FP8v1: AVGIGAVF**		**FP8v2: AVGLGAVF**		**FP8v3: AIGLGAMF**		**FP8v4:AVGTIGAMF**	**FP Thai: AVGIGAMI**			
	**Clade**	**A**	**ACD**	**AG**	**C**	**D**	**D**	**B**	**AE**	**AE**	**NA**	**NA**
	**Glycan**	complete	complete	complete	complete	complete	complete	625	complete	complete		
	**Week**	24	24	24	24	24	24	24	24	24		
FP8v1-rTTHC Monomer 11.2:1 Adjuplex	2431	35	37	13	8	6	5	7	13	45	5	2
	2432	28	**51**	19	0	17	9	6	19	**56**	2	12
	2433	25	26	18	5	17	13	14	23	31	16	14
	2434	28	37	21	3	41	41	13	**50**	**64**	14	35
	2435	21	29	14	12	19	16	3	23	20	7	9
	2436	**51**	47	27	22	11	22	19	30	**63**	19	17
	2437	42	36	13	-5	0	17	13	21	48	14	9
	2438	29	38	17	13	20	29	32	40	40	43	11
	2439	49	**57**	21	21	32	36	17	41	**65**	23	31
	2440	**55**	**56**	17	0	9	8	4	32	**72**	20	-1
FP8v1-rTTHC Multimer 4.8:1 Adjuplex	2401	**51**	**72**	12	-5	2	-5	15	-9	**50**	10	-4
	2402	46	28	7	-38	-9	-20	0	-7	38	-6	13
	2403	36	26	16	0	7	-6	16	-5	21	14	5
	2404	48	48	22	7	17	10	9	12	**69**	24	1
	2405	34	27	22	-15	19	19	10	10	47	13	14
	2406	6	6	16	-9	6	-1	9	-7	9	5	-15
	2407	43	**59**	21	20	18	25	6	18	**58**	26	14
	2408	48	**54**	20	3	10	8	7	9	**52**	21	17
	2409	29	46	14	2	12	4	-4	2	**55**	2	13
	2410	19	4	18	3	15	6	2	10	7	13	-13
FP8v1-rTTHC Multimer 4.8:1 Alum	2441	39	33	24	17	26	16	25	24	48	14	15
	2442	15	30	21	1	0	11	18	27	37	14	19
	2443	**54**	**65**	23	9	17	20	25	33	**53**	29	8
	2444	11	26	17	12	19	21	12	23	20	14	15
	2445	**54**	**56**	23	8	20	10	13	34	**56**	17	4
	2446	47	33	12	-7	6	10	7	16	**80**	10	9
	2447	36	**51**	22	14	14	19	13	30	**71**	16	16
	2448	45	**53**	16	5	15	21	18	34	41	23	4
	2449	**50**	**57**	31	29	37	34	20	41	6	33	8
	2450	45	40	22	-7	22	15	9	34	**66**	15	12

%neutralization	<50	50-59	60-69	70-79	80-89	90-100

Figure S6. Week 24 serum neutralization of 9 diverse FP heterologous wild-type strains. Sera were diluted 50 fold for the neutralization assay. Non-HIV viruses, SIVmac251 and SVA-MLV, were used as control. Entries with >50% neutralization were considered as positive.

Table S1.

FP8v1-rTTHC conjugation ratio calculated based on the amino acid analysis using Pro as internal standard

Pro IS						
Sample	Amino acids from FP8v1					Ave (with two outliers removed)
	Gly	Ala	Val	Ile	Phe	
FP8-rTTHC Control_TZ-863-114-05 (3 sample)	5.1	4.7	5.4	5.5	5.7	5.3
FP8v1-rTTHC, Lot# JV-723-089-09	5.6	3.3	5	5.3	5.3	5.2
FP8v1-rTTHC, Lot# JV-723-089-10	6.7	4.3	5.7	6.1	6.1	6.0
FP8v1-rTTHC, Lot# JV-723-089-11	6.8	6.3	7.3	7.6	7.5	7.2
FP8v1-rTTHC vrc40_04042019	10.9	11.1	9.7	11.9	7.8	10.6

FP8v1-rTTHC conjugation ratio calculated based on the amino acid analysis using Leu as internal standard

Leu IS						
Sample	Amino acids from FP8v1					Ave (with two outliers removed)
	Gly	Ala	Val	Ile	Phe	
FP8-rTTHC Control_TZ-863-114-05 (3 sample)	5.0	4.6	5.3	5.3	5.6	5.2
FP8v1-rTTHC, Lot# JV-723-089-09	5.5	3.2	4.8	4.9	5.1	4.9
FP8v1-rTTHC, Lot# JV-723-089-10	6.5	4.1	5.5	5.6	5.8	5.6
FP8v1-rTTHC, Lot# JV-723-089-11	6.8	6.3	7.3	7.5	7.4	7.2
FP8v1-rTTHC vrc40_04042019	10.4	10.6	9.2	10.8	7.3	10.1

FP8v1-rTTHC conjugation ratio calculated based on the amino acid analysis using Arg as internal standard

Arg IS						
Sample	Amino acids from FP8v1					Ave (with two outliers removed)
	Gly	Ala	Val	Ile	Phe	
FP8-rTTHC Control_TZ-863-114-05 (3 sample)	5.0	4.6	5.3	5.3	5.5	5.2
FP8v1-rTTHC, Lot# JV-723-089-09	5.0	2.3	4.3	3.7	4.6	4.2
FP8v1-rTTHC, Lot# JV-723-089-10	5.8	3.7	4.9	4.0	5.0	4.6
FP8v1-rTTHC, Lot# JV-723-089-11	6.9	6.4	7.4	7.8	7.6	7.3
FP8v1-rTTHC vrc40_04042019	13.6	13.3	12.2	17.8	10.6	13.0

FP8v1-rTTHC conjugation ratio calculated based on the amino acid analysis using Lys as internal standard

LYS IS						
Sample	Amino acids from FP8v1					Ave (with two outliers removed)
	Gly	Ala	Val	Ile	Phe	
FP8-rTTHC Control_TZ-863-114-05 (3 sample)	5.7	5.1	6	7.1	6.4	6.0
FP8v1-rTTHC, Lot# JV-723-089-09	7.3	4.4	6.5	9.5	7.3	7.0
FP8v1-rTTHC, Lot# JV-723-089-10	8.1	5.2	7.00	9.5	7.7	7.6
FP8v1-rTTHC, Lot# JV-723-089-11	7.3	6.7	7.8	8.8	8.1	7.7
FP8v1-rTTHC vrc40_04042019	7.1	7.9	6.1	3.4	3.8	5.7

FP8v1-rTTHC conjugation ratio

Conjugation Ratio/Average						
Sample	Lys IS	Pro IS	Arg IS	Leu IS	Ave (with one outlier removed)	% STD
FP8-rTTHC Control_TZ-863-114-05 (3 sample)	6.0	5.3	5.2	5.2	5.2	1.5
FP8v1-rTTHC, Lot# JV-723-089-09	7.0	5.2	4.2	4.9	4.8	10.8
FP8v1-rTTHC, Lot# JV-723-089-10	7.6	6.0	4.6	5.6	5.4	12.8
FP8v1-rTTHC, Lot# JV-723-089-11	7.7	7.2	7.3	7.2	7.2	1.0
FP8v1-rTTHC vrc40_04042019	5.7	10.6	13.0	10.1	11.2	14.2
